# Head‐To‐Head Comparison of Biologic Efficacy in Asthma: What Have We Learned?

**DOI:** 10.1111/all.16537

**Published:** 2025-03-29

**Authors:** Brian J. Lipworth, Robert Greig, Rory Chan, Chris RuiWen Kuo, Catherine Jackson

**Affiliations:** ^1^ Scottish Centre for Respiratory Research, Ninewells Hospital and Medical School, Department of Respiratory Medicine University of Dundee Dundee Scotland UK; ^2^ Department of Respiratory Medicine Aberdeen Royal Infirmary Aberdeen Scotland UK; ^3^ Department of Medicine and Health Sciences University of Lancashire Preston UK

**Keywords:** benralizumab, dupilumab, mepolizumab, omalizumab, tezepelumab

## Abstract

We performed an in‐depth appraisal of indirect head‐to‐head comparisons of biologics approved for asthma, including anti‐IL5/5Rα (mepolizumab, benralizumab), anti‐IL4Rα (dupilumab), anti‐TSLP (tezepelumab) and anti‐IgE (omalizumab), which was neither a systematic review nor a meta‐analysis. A crude evaluation of 95% CI's for rate ratios which excluded unity revealed greater overall reductions in annualised exacerbations with dupilumab versus either mepolizumab or benralizumab and also with tezepelumab versus benralizumab. Furthermore in patients with eosinophils ≥ 300/μL exacerbation rates were lower for tezepelumab, dupilumab and mepolizumab versus benralizumab; and with eosinophils< 150/μL for tezepelumab versus dupilumab. For lung function, no overall differences in FEV1 response were observed between drugs where there was considerable heterogeneity of overlapping 95% CI's. Dupilumab was superior to benralizumab for oscillometry‐derived peripheral lung resistance and compliance, as well as for attenuation of mannitol airway hyperresponsiveness. There were no differences in asthma control or quality of life scores where the effect sizes were small, along with wide overlaps in 95% CI's. There is an unmet need for prospective pragmatic randomised controlled trials to directly compare biologics, especially to assess clinical remission in both type 2 high and low asthma patients. Real‐life studies might also evaluate complete remission with different biologics to include outcomes such as inhaled corticosteroid sparing, small airways dysfunction using oscillometry, abolition of airway hyperresponsiveness and to assess mucus plugging and remodelling as wall thickening with imaging.

**Future Research Perspectives**

- Prospective pragmatic randomized controlled trials are indicated to directly compare biologics in severe uncontrolled asthma powered on the propensity to achieve clinical remission.
- Such prospective studies might require collaboration between independent funding bodies and the pharmaceutical industry.
- A alternative approach for a pooled re‐analysis of data from existing phase 3 randomized controlled trials might usefully employ win ratios to indirectly compare biologics looking at hierarchical composite end points.
- Prospective real life studies could also explore the possibility for different biologics to produce complete clinical remission including outcomes such as inhaled corticosteroid sparing, abolition of airway hyperresponsiveness, improvement in small airways dysfunction using oscillometry, as well as attenuation of mucus plugs and remodeling as airway wall thickness with imaging.
- Such studies could be performed in patients with triple type 2 high or low biomarker phenotypes.
- It will be especially interesting to see if bispecific biologics such as lunsekimib blocking dual signaling pathways of TSLP and IL13 may prove to be more effective than respective mono‐specific blockers in improving disease control.

## 1 Introduction

**FIGURE 1:**
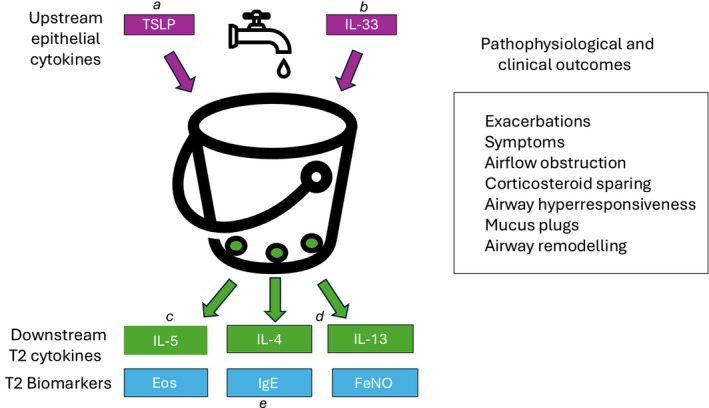
Schematic diagram simplified to depict the type 2 immunology pathway in asthma. The upstream epithelial cytokine tap drips TSLP and IL33, which activate downstream T2 cytokines to fill the mucosal airway bucket with escape of IL4/5/13. The epithelial cytokines may have pathological effects in their own right aside from promoting activation of downstream T2 cytokines. IgE release may be mediated via both IL4 and IL13. The T2 cascade may be blocked either upstream by (a) anti‐TSLP as tezepelumab, (b) anti‐IL33 as itepekimab; or downstream by (c) anti‐IL5/IL5Rα, as mepolizumab or benralizumab, (d) anti‐IL4Rα as dupilumab, (e) anti‐IgE as omalizumab. Eos, eosinophils; FeNO, fractional exhaled nitric oxide; IgE, immunoglobulin E; IL, interleukin 4/5/13/33; T2, type 2 inflammation; TSLP, thymic stromal lymphopoietin.

The introduction of biologic monoclonal antibody drugs has revolutionised the treatment of severe asthma, especially for those with the refractory type 2 (T2) high inflammatory phenotype. Initially this involved using omalizumab (Omal) as anti‐immunoglobulin‐E (IgE), followed by T2 cytokine blockers including mepolizumab (Mepo) and reslizumab (Resli) as anti‐interleukin‐5 (IL5), benralizumab (Benra) as anti‐IL5 receptor alpha (IL5Rα) and dupilumab (Dupi) as anti‐IL4 receptor alpha (IL4Rα) [1]. Latterly biologics have become available which block epithelial cytokines (alarmins) such as thymic stromal lymphopoietin (TSLP) with tezepelumab (Teze) [2]. These biologic agents act on different parts of the T2 inflammatory pathway (Figure 1) on either upstream or downstream proinflammatory cytokines, to exhibit their anti‐asthmatic clinical efficacy in patients who are uncontrolled despite conventional dual or triple combination therapy as inhaled corticosteroid with long‐acting beta‐agonist (ICS/LABA) or with long‐acting muscarinic antagonist (ICS/LABA/LAMA), along with leucotriene receptor antagonist (LTRA). Current guidelines advocate the use of biologic drugs as add‐on therapy at step 5 in uncontrolled frequently exacerbating asthma patients including those requiring maintenance systemic corticosteroids (SCS) [3]. The choice of such biologic therapy should be tailored according to prevailing T2 biomarkers including blood eosinophils (Eos), fractional exhaled nitric oxide (FeNO) and IgE, as well as other phenotypic characteristics (Figure 1).

The aim of the present article was to critically appraise the current literature with regard to indirect head‐to‐head (H2H) comparisons of commonly used biologics for patients with severe uncontrolled asthma.

## 2 Type 2 Immunology and Biomarkers

The pathophysiology and immunology of the type 2 low and high inflammatory pathway along with the effects of biologics have been reviewed in detail elsewhere [4]. In essence, maturation of eosinophils in the bone marrow and their migration via blood is mediated by IL5, while eosinophil transit from the blood into the lungs is in turn controlled by IL4 and IL13 [5]. FeNO levels are a reflection of IL13 expression, while IgE levels reflect expression of IL4 and IL13 [6, 7, 8]. While the level of total IgE is used to adjust the dose of Omal, suppression of IgE may be considered a desirable therapeutic effect when selecting a biologic such as Dupi or Teze. This might be the case, for example, in those patients who have levels of IgE higher than approved for using Omal or in patients who have concomitant atopic disease such as allergic rhino‐conjunctivitis.

The levels of blood eosinophils and FeNO, when combined together, predict the exacerbation risk [9]. A simplified but pragmatic analogy of T2 biology is depicted in Figure 1 which comprises a downstream mucosal inflammatory cytokine bucket with three holes, out of which the cytokines IL4, IL5 and IL13 may escape. The bucket is filled by the upstream epithelial cytokine tap, which constantly drips either TSLP or IL33 [10]. These epithelial alarmins may have pathological effects in their own right, aside from activating downstream T2 cytokine expression. In terms of clinical biomarkers, Mepo and Benra will reduce blood eosinophils, Dupi will lower FeNO and IgE levels, while Teze will suppress all three biomarkers.

Plugging the downstream IL5 leak with anti‐IL5/5Rα as Mepo or Benra will attenuate eosinophilic inflammation but will leave persistent escape of IL4 and IL13 and associated elevated levels of IgE and FeNO [11, 12]. For example, one real‐life study looked at patients with severe eosinophilic asthma who had previously failed on Mepo or Benra and were then switched to Dupi, where over 2 years of subsequent follow‐up, there was a 40% clinical remission associated with higher prebiologic FeNO levels, in keeping with a predominant endotype characterized by IL13 signalling [13]. Thus, anti‐IL5/5Rα agents can be considered as exhibiting a rather narrow spectrum of anti‐inflammatory activity in patients with T2 high asthma.

Anti‐IL4Rα therapy as Dupi plugs the leakage of IL4 and IL13, resulting in reduced levels of IgE and FeNO, along with a rise in blood eosinophils due to the prevention of eosinophil trafficking from the blood into the lung [14, 15]. Usually, this eosinophil rise is transient due to equilibration of eosinophil formation between the blood and bone marrow [16, 17]. Since Teze turns off the epithelial TSLP tap, this prevents the T2 bucket from filling up with T2 cytokines and therefore reduces levels of eosinophils, IgE and FeNO [18]. Hence, both Teze and Dupi can be considered as being more broad spectrum in their anti‐inflammatory profiles.

The main difference between Teze and Dupi is that the latter may be associated with eosinophil escape in the blood, which in rare cases can potentially cause hypereosinophilia [16] and uncover the presence of underlying eosinophilic granulomatosis with polyangiitis (EGPA). Moreover, in the presence of anti‐TSLP with Teze, there may be unopposed epithelial IL33 expression with associated partial escape of downstream T2 cytokines and raised T2 biomarkers, which was evident in the attenuation of downstream T2 biomarkers by the anti‐IL33 drug itepekimab [19].

Anti‐IgE therapy as Omal acts further down the T2 cascade on IgE, resulting in a narrower spectrum of anti‐inflammatory activity, and therefore reductions in T2 biomarkers while taking Omal are modest [20]. Patients with higher eosinophils and FeNO at baseline tend to fare better in terms of exacerbation reductions with Omal [21]. While the anti‐IL5 drug Mepo produces consistent reductions of blood eosinophils, its effects on airway eosinophils are more variable. The 100 mg dose of Mepo, which is approved for asthma, may be considered suboptimal given that 300 mg is approved for EGPA, where eosinophil levels are much higher [22, 23, 24]. The approved dose of Berna is 30 mg every month for EGPA and every 2 months for asthma [25]. The anti‐IL5Rα agent Benra produces almost complete and rapid homogeneous depletion of eosinophils in both the blood and airway, which might, in theory, be expected to translate into a better clinical efficacy, especially in patients who have higher baseline levels of blood eosinophils [12, 26, 27]. It is worth noting that in patients taking Berna, there may be a relative disconnect between the suppression of blood and airway eosinophils and persistently raised FeNO. One hypothesis regarding the heterogeneity of eosinophil suppression is the concept of preserving so‐called homeostatic eosinophils in the presence of partial suppression by Mepo, although this putative concept remains unproven in terms of clinical efficacy outcomes [28].

Raised FeNO levels due to unopposed IL13 signalling seen in patients treated with antiIL5/Rα may also be a reflection of poor adherence to ICS containing therapy [29, 30, 31], while elevated levels of FeNO prior to taking Mepo or Berna may be associated with worse disease control [13]. In this regard, in the presence of ICS sparing using maintenance and reliever therapy (MART), levels of FeNO remain suppressed while taking Dupi [17] but may increase with Benra [32]. Having said that, these two studies had inherently different designs and hence it is not possible to infer any comparison between Dupi and Benra in regards to ICS sparing potential. In a Scottish cohort of moderate to severe asthma patients, there was considerable overlap of T2 biomarkers according to eosinophils ≥ 300/μL, FeNO ≥ 25 ppb, and total IgE ≥ 100 kU/L, where 24.7% of patients were classified as being triple T2 high and 18.5% triple T2 low [33]. Here, the triple T2 high signature in turn conferred more frequent exacerbations and worse lung function compared to those who were triple low. In an international severe asthma registry study, 27% of patients had the triple T2 high phenotype while 12% were triple T2 low [34].

## 3 Clinical Efficacy of Biologics in Asthma

The main clinical impact of biologics in T2 high asthma is to reduce exacerbation frequency and ameliorate symptoms assessed with the asthma control questionnaire (ACQ), improve airflow obstruction assessed by lung function and obviate the use of systemic corticosteroid (SCS) either as rescue or maintenance therapy (Figure 1) [2]. These desired clinical outcomes comprise the definition of either a so‐called early super responder or more sustained clinical remission [35, 36]. It has been proposed for the term complete remission to be used to include ancillary effects of biologics on other key phenotypic characteristics, including attenuation of airway hyperresponsiveness (AHR), small airways dysfunction (SAD), mucus plugging and airway remodelling [35, 37, 38](Figure 1). Suppression of type 2 biomarkers has also been proposed as a possible outcome in defining complete remission. However, this concept is inherently flawed as it is possible to achieve clinical remission in relation to annualised exacerbation rate (AER), ACQ and SCS with Mepo or Benra without suppressing FeNO, or by the same token with Dupi without suppressing blood eosinophils. Other factors, including comorbidities, should be taken into account in selecting the most appropriate drug, for example, treating the whole airway in patients with uncontrolled asthma with chronic rhinosinusitis with nasal polyps, where biologics have varying levels of efficacy [39, 40].

**FIGURE 2:**
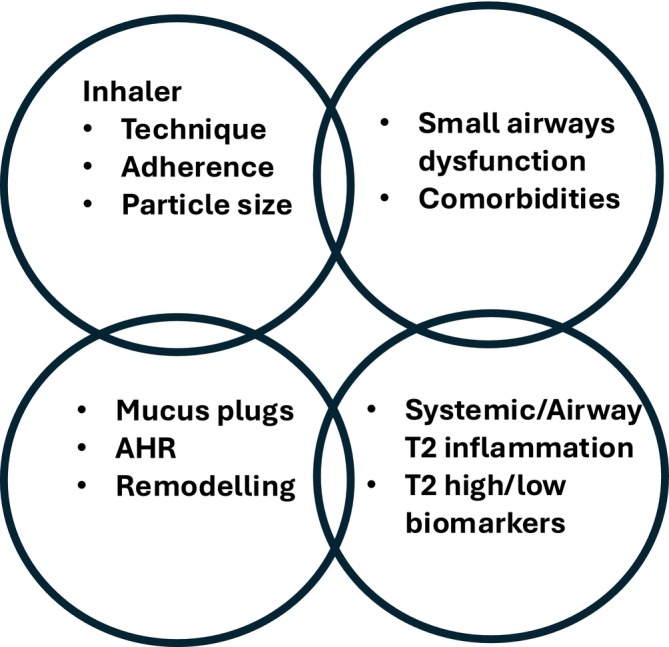
Schematic diagram to illustrate the interplay of various factors which may determine the efficacy of biologics in refractory severe asthma. AHR, airway hyperresponsiveness; T2, type 2 inflammation.

There are several cogent reasons as to why biologics may improve control in severe refractory asthma (Figure 2). For example, the systemic route of administration is likely to explain the anti‐asthmatic efficacy of biologics in patients who are unable to use their inhalers properly or are nonadherent, along with poor penetration of delivered particles > 2 um into narrowed peripheral airways [38, 41]. The systemic route will be able to reach mucosal T2 inflammation throughout the entire lung, as well as treat inflammatory cells and their progenitors in the bone marrow and blood. Other factors such as comorbidities may also determine the response to biologics, which are out with the scope of this review.

Current asthma management guidelines recommend choosing initial biologic therapy according to the prevailing T2 biomarkers and other phenotypic manifestations and then, in the absence of either super response and/or clinical remission, to consider switching to another agent in sequential fashion [3]. Using combination biologic therapy to effectively block the T2 inflammatory pathway, although logical from a mechanistic viewpoint [14] is in reality prohibitively expensive for real‐life clinical practice. Hence, within such fiscal constraints, it is important for clinicians to have an understanding of how the various biologics might perform on a putative head to head (H2H) basis in order to select the right drug for the right patient in order to optimise the clinical response and avoid having to switch between drugs [42]. Ideally, there would be prospective H2H randomised clinical trials (RCT) to guide optimal biologic prescribing, although in reality, this type of data does not currently exist due to the reluctance of the pharmaceutical industry to take on board the inherent risks of performing such studies. The PREDICTUMAB trial is an independent Belgian pragmatic prospective comparison of Mepo versus Omal in adults with allergic eosinophilic asthma (ClinicalTrials.gov NCT03476109) looking at predictive factors and magnitude of response. The CHOOSEBETWEENAMAB trial from Australia also compares MEPO versus OMAL in allergic eosinophilic asthma with randomisation stratified by blood eosinophil count (ClinicalTrials.gov ID NCT04585997) which was due to complete by 2022.

Thus, at present, the most robust data have been gleaned from appraising indirect head‐to‐head comparisons of different biologics from either phase 2 or phase 3 randomised controlled trials (RCT) or from real‐life health informatics data. In this regard, the numerous systematic reviews and associated meta‐analyses which have been performed to indirectly compare biologics are often driven by pharmaceutical companies who may have vested interests in publishing the right result for their particular drug.

## 4 Methodology

There is an unmet need in the literature to perform a critical appraisal of the available literature which have indirectly compared the various biologics to try and synthesise the data and draw some conclusions regarding their relative clinical efficacy in patients with uncontrolled asthma. We decided therefore to restrict our appraisal to those studies which have published robust methodology for H2H data analysis in adults with uncontrolled asthma, excluding preliminary abstracted data which have not been properly peer reviewed. PubMed, Embase and Scholar were used to search for eligible studies for inclusion using appropriate search terms.

In terms of synthesising the available data, there was a focus on appraising key clinical outcomes including rate ratios (RR) and 95% confidence intervals (95% CI) for annual exacerbation rate (AER) and SCS sparing activity, where these were quoted for indirectly comparing effects of biologics on a H2H basis and only where it was possible to identify the specific drug rather than a generic class of biologic, such as pooling of anti‐IL5 with anti‐IL5Rα.

In addition, data were extracted where available for H2H comparisons for absolute differences in ACQ score and asthma quality of life (AQLQ) score. Effects on airflow obstruction were evaluated either as spirometry‐derived forced expiratory volume in 1 s (FEV1) as well as oscillometry‐derived peripheral lung resistance as heterogeneity between 5 and 20 Hz (R5–R20) and peripheral lung compliance as area under the reactance curve (AX). We have also appraised some real‐life studies which have compared biologics H2H, although this article will not include real‐life biologic switching studies where there is an inherent sequential bias incurred as a result of having to initially fail on one particular drug before starting another, where there is also likely to be a confounding carryover effect.

Pointedly, this article is not intended to be a systematic review or a meta‐analysis of the various biologics, as this has already been done, albeit with different methodologies. For the various Forest plots, we did not factor in sample size weighting or heterogeneity analysis, and no pooled estimate of the overall effect size was calculated, given that the H2H comparisons were made between different biologics and not versus placebo. Nonetheless, the Forest plots provide a way to assess which of the H2H comparisons are significant in terms of crude inspection of the 95% CI. A pragmatic decision was made to only include the commonly used and approved biologics comprising Omal, Mepo, Benra, Dupi, and Teze, but not reslizumab, which is rarely prescribed due to practical and fiscal issues related to its intravenous administration.

## 5 Asthma Exacerbations

Given that the pivotal phase 3 registration placebo‐controlled RCT's were powered on AER there are a plethora of data indirectly comparing different biologics from individual studies where a RR has been calculated for relative reduction in exacerbations between pairs of drugs. Having said that it is worth mentioning that although enrolled patients in these various RCT's were required to have poorly controlled asthma, there are some inherent differences in the prevailing exacerbation rates prior to enrolment as well as the AER in the placebo arms of the RCT's. By definition patients who were enrolled with poorly controlled asthma tend to be those with T2 high disease in terms of having elevated levels of either eosinophils, FeNO and IgE at baseline, usually taking high dose ICS containing combination therapy as well as a requirement for maintenance oral corticosteroids (mOCS). Although the baseline phenotypes might vary between RCTs, these putative differences have usually been taken into account as confounding co‐factors in the analysis model.

**FIGURE 3:**
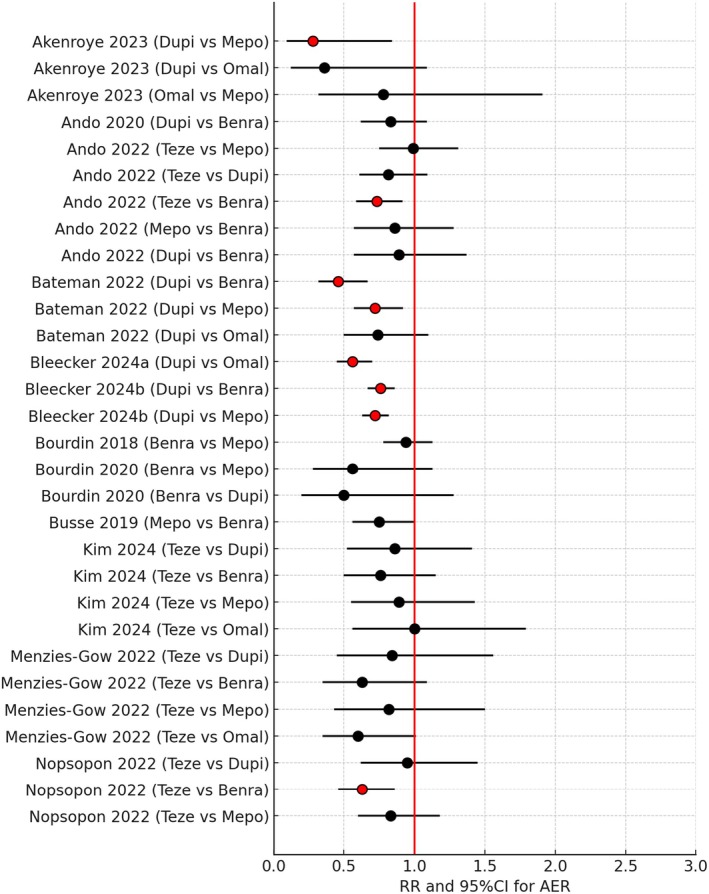
Forest plot showing pairwise indirect comparisons of biologics in uncontrolled asthma for reductions in overall annualised exacerbation rates (AER) shown as rate ratios (RR) and 95% confidence intervals (CI). For crude pairwise comparisons of drug A versus drug B, red circles for the RR denote a significant difference in favour of drug A where the 95% CI excludes unity. Black circles for the RR denote no significant difference between biologics where the 95% CI includes unity [43, 45, 46, 47, 48, 49, 50, 51, 52, 54, 55, 56]. Benra, benralizumab; Dupi, dupilumab; Mepo, mepolizumab; Omal; omalizumab; Teze, tezepelumab.

A list of studies which met the above criteria in terms of reporting the AER are given in Table 1 along with the overall outcome comparing different biologics [43, 44, 45, 46, 47, 48, 49, 50, 51, 52, 53, 54, 55, 56]. A more granular analysis of relative RR and 95% CI comparing overall AER for biologics is also depicted as a Forest plot in Figure 3 [43, 45, 46, 47, 48, 49, 50, 51, 52, 54, 55, 56]. Here, the RR and 95% CI for H2H overall comparisons irrespective of T2 biomarkers between drugs are given but not comparisons versus placebo per se.

**TABLE 1:** Studies which have indirectly compared biologics in patients with uncontrolled asthma with regard to their effects on exacerbations as the primary outcome [43, 44, 45, 46, 47, 48, 49, 50, 51, 52, 53, 54, 55, 56].

Author	Year	Study	Biologics	Outcomes	AER effect
Akenroye *N* = 201	2023	Target trial emulation	Dupi, Omal, Mepl	AER, FEV1	Dupi > Mepo
Al‐Shaikhly *N* = 5538	2024	US claims	Dupi, Mepo, Benra, Omal	AER	Dupi > Mepo/Benra/Omal
Ando *N* = 2460	2020	Indirect comparison	Dupi, Benra	AER, AQLQ, FEV1	Dupi > Berna (Eos > 300/μL)
Ando *N* = 5524	2022	Network meta	Teze, Dupi Mepo, Benra	AER, AQLQ, ACQ, FEV1	Teze > Benra Teze > Dupi (Eos < 150/μL)
Bateman *N* = 3459	2022	Indirect comparison	Dupil, Benra Mepo, Omal	AER, FEV1	Dupi > Mepo/Benra/Omal
Bleecker *N* = 3451	2024a	US real‐world	Dupi, Omal	AER, SCS	Dupi > Omal
Bleecker *N* = 1737	2024b	US real‐world	Dupi, Benra, Mepo	AER, SCS	Dupi > Mepo/Benra
Bourdin *N* = 2423	2018	Matching indirect comparison	Benra, Mepo	AER, FEV1	Mepo = Benra
Bourdin *N* = 493	2020	Matching indirect comparison	Benra, Mepo, Dupi	AER, SCS	Dupi = Mepo = Benra
Busse *N* = 1127	2019	Indirect comparison	Mepo, Benra	AER, ACQ, FEV1	Mepo > Benra
Iftikhar *N* = 8444	2018	Network meta	Dupi, Mepo, Benra	AER, FEV1, AQLQ, ACQ	Dupi = Mepo = Benra
Kim *N* = 8376	2024	Network meta	Teze, Dupi, Mepo, Benra	AER, QLQ, ACQ, FEV1	Teze = Dupi = Mepo = Benra
Menzies‐Gow *N* = 9139	2022	Indirect comparison	Teze, Dupi, Mepo, Benra, Omal	AER	Teze = Dupi = Mepo = Benra = Omal
Nopsopon *N* = 9201	2023	Bayesian network meta	Teze, Dupi, Benra, Mepo	AER, ACQ, FEV1	Teze > Benra

Abbreviations: ACQ, asthma control questionnaire; AER, annualised exacerbation rate; AQLQ, asthma quality of life questionnaire; Benra, benralizumab; Dupi, dupilumab; FEV1, forced expiratory volume in 1 s; Mepo, Mepolizumab; Meta, meta‐analysis; Omal, omalizumab; SCS, systemic corticosteroid; Teze, tezepelumab.

On inspecting the Forest plot in Figure 3 some clear patterns emerged worthy of note. First, 22/30 (73%) of the individual H2H indirect crude comparisons were not significant in terms of the 95% CI for AER including unity, while 8/30 (27%) of comparisons were significantly different with the 95% CI excluding unity. Of those that were significantly different 2/8 were in favour of Teze and 5/8 were in favour of Dupi, ≥≥in comparison to anti‐IL5/5Rα. Moreover there were 4 comparisons between Teze and Dupi which were not significantly different where there was also considerable overlap of the respective 95% CI. One possible conclusion from this composite Forest plot is that there is considerable variability in response between different biologics when indirectly comparing their relative efficacy for reducing exacerbations. This, in turn, is likely to reflect that most frequently exacerbating patients enrolled in phase 3 studies are inherently T2 high, such that all biologics confer a reasonable level of clinical efficacy.

**FIGURE 4:**
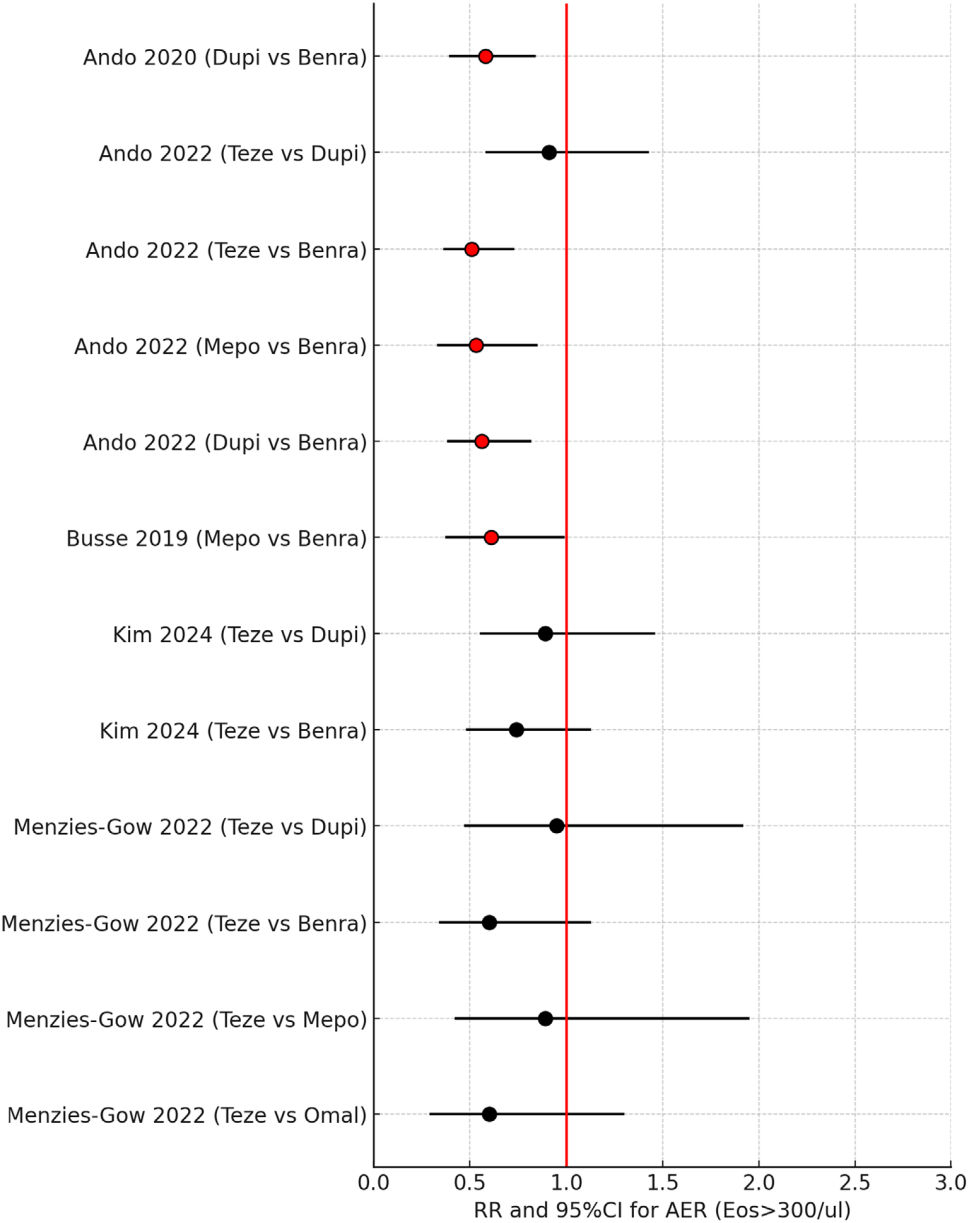
Forest plot showing pairwise indirect comparisons of biologics in uncontrolled asthma for reductions in annualised exacerbation rates (AER) for the subgroup of patients with baseline eosinophils (Eos) ≥ 300/μL, as rate ratios (RR) and 95% confidence intervals (CI). For crude pairwise comparisons of drug A versus drug B, red circles for the RR denote a significant difference in favour of drug A where the 95% CI excludes unity. Black circles for the RR denote no significant difference between biologics where the 95% CI includes unity [45, 46, 52, 54, 55]. Benra, benralizumab; Dupi, dupilumab; Mepo, mepolizumab; Omal, omalizumab, Teze, tezepelumab.

It is nonetheless also worth commenting on studies which have also looked in more depth at effects on AER according to T2 biomarkers. A Forest plot for H2H comparisons according to eosinophils ≥ 300/μL for AER is shown in Figure 4 comprising 12 H2H drug comparisons [45, 46, 52, 54, 55]. There were 5/12 (42%) of H2H comparisons for eosinophils ≥ 300/μL which were significantly different where the 95% CI excluded unity, indicating worse outcomes with Benra in such T2 high patients.

In particular, it is worth focusing on the results from a network meta‐analysis by Ando et al. [46] which notably received no external pharmaceutical funding and had no associated reported conflicts of interest, indirectly comparing AER's with Teze, Mepo, Benra and Dupi in 5524 patients with uncontrolled asthma. A subgroup analysis of AER according to T2 biomarkers at baseline for Teze versus Dupi in patients with eosinophils ≥ 300/μL revealed a RR of 0.91 (95% CI 0.58 to 1.43) and 0.53 (95% CI 0.30 to 0.94) in patients with eosinophils < 150/μL, indicating 47% significantly fewer annual exacerbations in response to Teze in T2 low patients. Interestingly, for the same biologic comparison, there were no significant differences in RR's for AER in respect of either T2 high as FeNO ≥ 50 ppb or T2 low as FeNO < 25 ppb, with corresponding RR's of 0.87 (95% CI 0.49 to 1.57) and 0.86 (95% CI 0.55 to 1.09). Here, when comparing Teze versus Benra, there was a significant difference in AER with eosinophils ≥ 300/μL, but not with eosinophils < 150/μL where respective RRs were 0.51 (95% CI 0.36 to 0.73) and 0.85 (95% CI 0.50 to 1.44). The findings of Ando et al. [46] with regard to superiority with Teze versus Benra in T2H patients are perhaps surprising, given that only partial suppression of blood and airway eosinophils occurs with the former. In this regard, a pooled analysis of two phase 3 trials with Teze verus placebo showed greater effects on AER in T2 high versus T2 low patients, along with a more heterogeneous response in the latter [57].

Ando et al. also reported a significantly greater AER reduction with Mepo versus Benra in association with Eos ≥ 300/μL with a RR of 0.53 (95% CI 0.33 to 0.85), indicating 47% fewer annual exacerbations with Mepo [46]. This is also somewhat counterintuitive given that Mepo partially suppresses blood and airway eosinophils compared to Benra [26, 27, 58]. Likewise, there was a significant difference in AER with Eos ≥ 300/μL for Dupi versus Benra as a RR of 0.56 (95% CI 0.38 to 0.82), where there were 44% fewer exacerbations with Dupi [46]. These observations, in turn, perhaps challenge the concept that Benra might be the logical first choice for patients with higher levels of eosinophils at baseline.

It is also worth looking at real‐world evidence data for exacerbations where responses may differ from the setting of a RCT due to somewhat artificial enrolment criteria involved in the latter. One analysis by Al‐Shaikhly [44] of US claim‐based data from 5538 patients taking biologics found that compared to Dupi as a reference, the likelihood of experiencing ≥ 2 exacerbations was 52% (95% CI 34 to 65) higher with Benra,78% (95% CI 71 to 84) higher with Mepo, and 76% (95% CI 69 to 81) higher with Omal. A meta‐analysis of 21 real‐life studies by Charles et al. [59] reported no difference between Berna and Mepo where there was wide overlap between the 95% CI for absolute AER reduction, which were, respectively, −3.79 (95% CI −4.53 to −3.04) versus. −3.17 (95% CI −3.74 to −2.59), although a relative rate ratio was not provided. Thomas et al. [60] evaluated 453 patients taking Mepo and Omal from two real‐world Australian severe asthma registries where clinical remission occurred in 29.3% of patients for Mepo and 22.8% for Omal. In another real‐life study from Spain of 410 patients, there were 19.6% in clinical remission with Mepo, 25.8% with Berna, and 31.6% with Omal [61]. No formal comparisons between drugs were made in either study where there were apparent differences in baseline biomarkers and other phenotypic characteristics. In an open‐label study of severe eosinophilic asthma patients suboptimally controlled on Omal who were switched to Mepo, there was a 64% subsequent reduction in AER [62].

A prospective phase 2 RCT comparing anti‐IL33 with itepekimab, anti‐IL4Rα with Dupi, either alone or in combination versus placebo, was performed in moderate to severe asthma patients who underwent ICS tapering. The odds ratios for loss of control versus placebo were 0.33 (95% CI 0.15 to 0.70) for Dupi, 0.42 (95% CI 0.20 to 0.88) for itepekimab and 0.52 (95% CI, 0.26 to 1.06) for the combination, indicating no additivity of response when blocking IL33 in addition to IL4/13 [19]. In this regard, it will be especially interesting to see if bispecific antibodies such as anti‐TSLP/anti‐IL13 (Sanofi, Lunsekimig) may confer any synergy of response [63, 64].

## 6 Systemic Corticosteroid Sparing

**FIGURE 5:**
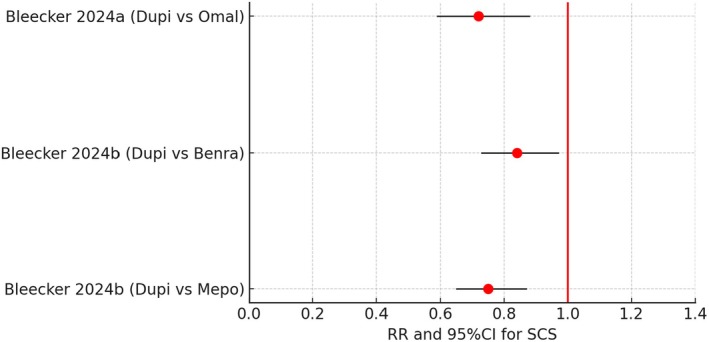
Forest plot showing pairwise indirect comparisons of biologics in uncontrolled asthma for reductions in use of systemic corticosteroid (SCS), as rate ratios (RR) and 95% confidence intervals (CI). For crude pairwise comparisons of drug A versus drug B, red circles for the RR indicate a significant difference in favour of drug A where the 95% CI excludes unity [48, 49]. Benra, benralizumab; Dupi, dupilumab; Mepo, mepolizumab; Omal, omalizumab.

There were only 3 evaluable H2H comparisons for oral corticosteroid (OCS) sparing effects of biologics when expressed as rate ratios (Figure 5) [48, 49]. These all showed a significant reduction in OCS exposure when comparing Dupi versus either Mepo, Benra, or Omal. A case matched adjusted comparison of OCS burden by Bourdin [51] reported a mean 6.1% (95% CI −22.2 to 34.4) difference for OCS dose reduction between Berna and Mepo, with the corresponding difference for Benra versus Dupi being −0.7% (95% CI −20.6 to 19.2). For OCS elimination the odds ratios were 2.3 (95% CI 0.5 to 11.5) and 2.3 (95% CI 0.5 to 9.8) for Benra versus Mepo and Benra versus Dupi respectively. Taken together, these data indicate that there was no significant difference in propensity for OCS sparing among the three biologics.

## 7 Asthma Control and Quality of Life

**FIGURE 6:**
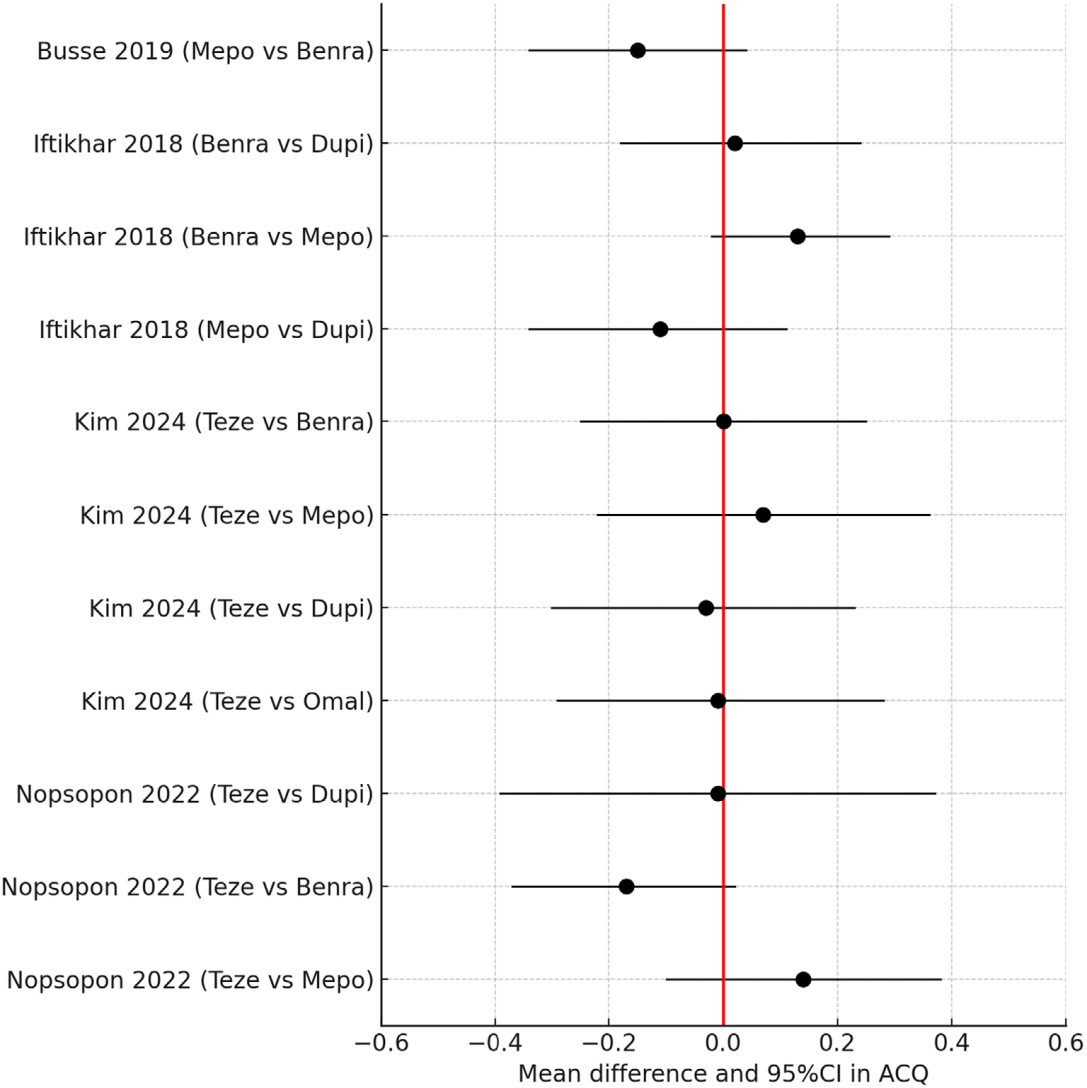
Forest plot showing pairwise indirect comparisons of biologics in uncontrolled asthma for reductions in asthma control questionnaire (ACQ) score as mean difference and 95% confidence intervals (CI). For pairwise comparisons of drug A versus drug B, a negative value for the difference indicates a greater improvement for drug A versus drug B. Black circles for RR denote no significant difference between biologics where the 95% CI includes zero. The minimal importance difference is +/−0.5 [52, 53, 54, 56]. Benra, benralizumab; Dupi, dupilumab; Mepo, mepolizumab; Omal, omalizumab; Teze, tezepelumab.

Those studies which reported H2H comparisons in ACQ scores are given in Figure 6 which showed considerable overlap of the 95% CI [52, 53, 54, 56]. There were no significant differences between any of the biologics for the change in ACQ scores since all of the 95% CI included zero, while the 95% CI were contained within the MCID of ±0.5 [65]. No analysis was available to assess the relative proportion of patients who had absolute ACQ scores < 1.5 which is the cut‐off for poor disease control. The ACQ is highly relevant since an absolute score > 1.5 is highly predictive of the risk of a future exacerbation [66, 67] while a score < 1.5 is used as part of the definition for a super responder or clinical remission.

**FIGURE 7:**
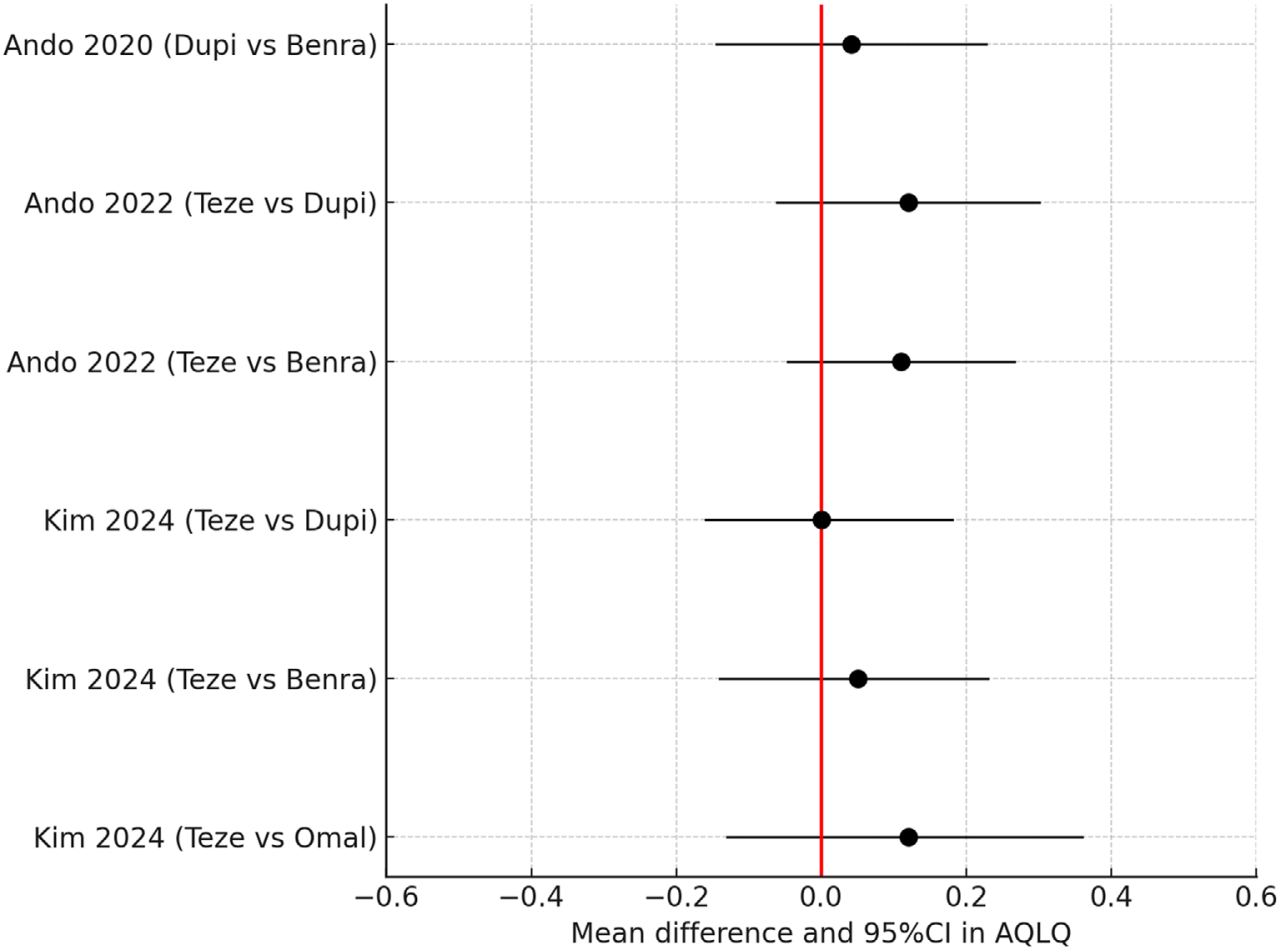
Forest plot showing pairwise indirect comparisons of biologics in uncontrolled asthma for reductions in asthma quality of life questionnaire (AQLQ) score as mean difference and 95% confidence intervals (CI). For pairwise comparisons of drug A versus drug B, a positive value for the difference indicates greater improvement for drug A versus drug B. Black circles for RR denote no significant difference between biologics where the 95% CI includes zero. The minimal importance difference is +/−0.5 [45, 46, 54]. Benra, benralizumab; Dupi: Dupilumab, Mepo, mepolizumab; Omal, omalizumab; Teze, tezepelumab.

A similar pattern emerged for AQLQ scores with no differences between biologics and the 95% CI were contained within the MCID of +/−0.5 [68] (Figure 7) [45, 46, 54]. However, AQLQ scores are not conventionally used to define either super responders or clinical remission. It is evident from most of the phase 3 RCT's that biologics have a lesser impact on ACQ and AQLQ than on AER, which may explain why there were no observed differences in response for H2H comparisons. Indeed, none of these phase 3 studies was powered on either ACQ or AQLQ per se.

## 8 Airflow Obstruction

**FIGURE 8:**
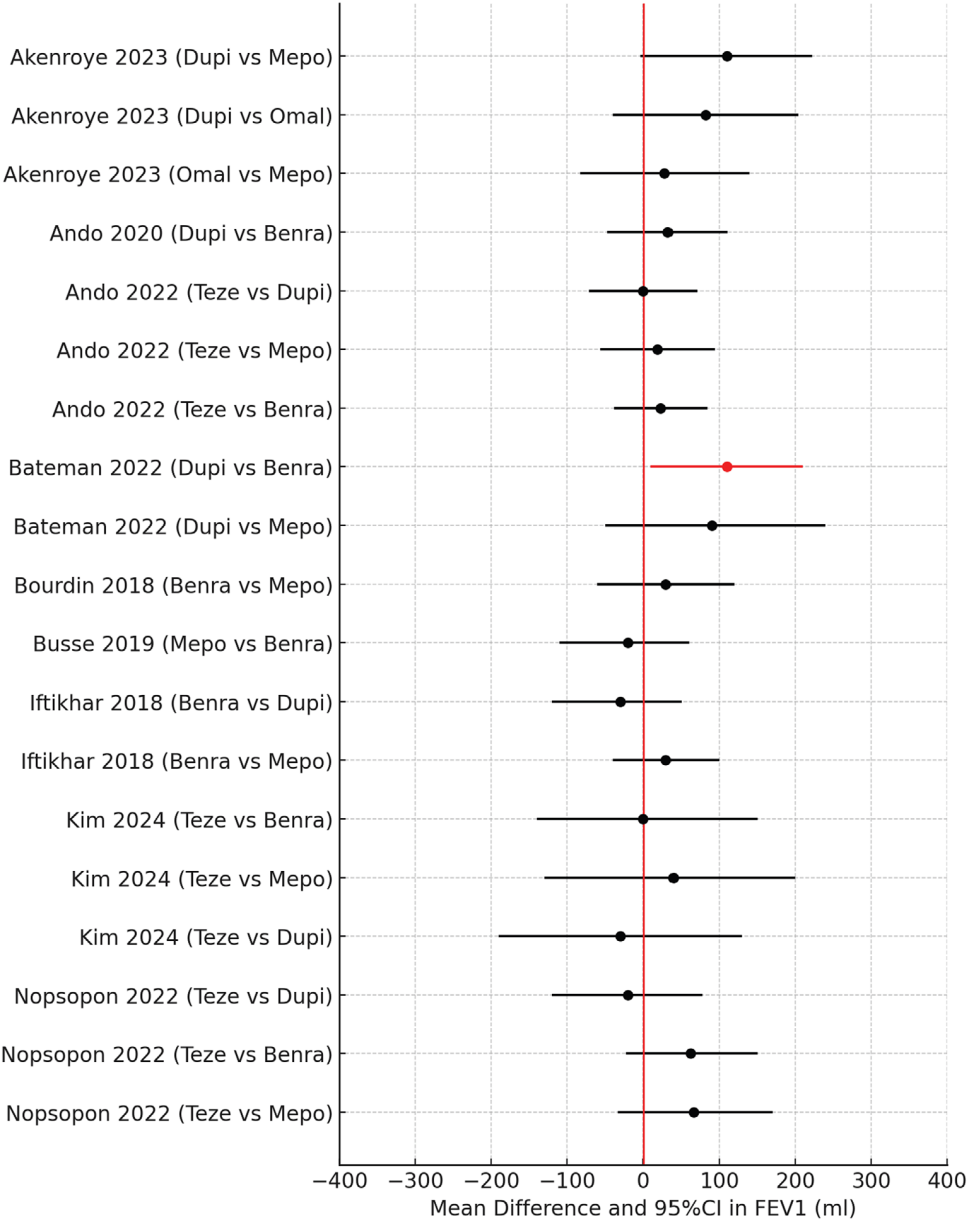
Forest plot showing pairwise indirect comparisons of biologics in uncontrolled asthma for overall improvements in FEV1 as mean difference and 95% confidence intervals (CI). For crude pairwise comparisons of drug A versus drug B, red circles denote a significant difference in favour of drug A where the 95% CI excludes zero, and black circles denote no significant difference between biologics where the 95% CI includes zero. The minimal importance difference for FEV1 is +/−150 mL [43, 45, 46, 47, 50, 52, 53, 54, 56]. Benra, benralizumab; Dupi, dupilumab; Mepo, mepolizumab; Omal, omalizumab; Teze, tezepelumab.

As a rule, biologics tend to exhibit only modest improvements in lung function, especially with respect to effort‐dependent spirometry outcomes such as FEV1, which reflects larger airways > 2 mm in calibre up to generation 8 of the bronchial tree. There was considerable overlap between the 95% CI for the 19 H2H comparisons of FEV1 (Figure 8) [43, 45, 46, 47, 50, 52, 53, 54, 56]. Only one of these studies by Bateman 2022 [47] showed a significant difference in favour of Dupi versus Benra for FEV1, with the 95% CI excluding zero, although the confidence interval was wide. However, the same pairwise comparison reported by Ando [45] did not show any difference, casting some doubt on the validity of the finding.

**FIGURE 9:**
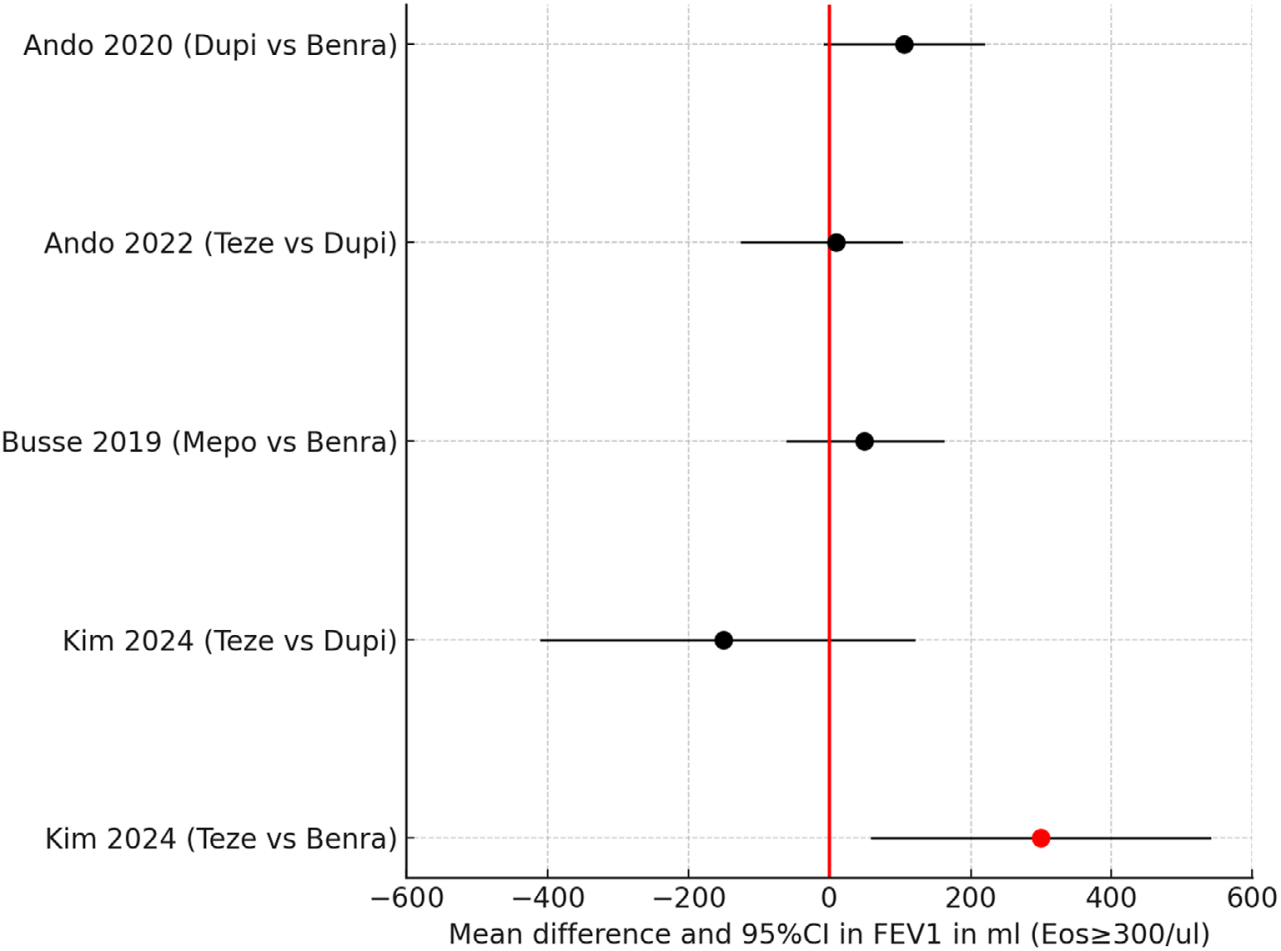
Forest plot showing pairwise indirect comparisons of biologics in uncontrolled asthma for improvements in FEV1 for the subgroup of patients with baseline eosinophils (Eos) ≥ 300/μL as mean difference and 95% confidence intervals (CI). For pairwise comparisons of drug A versus drug B, red circles denote a significant difference in favour of drug A where the 95% CI excludes zero, and black circles denote no significant difference between biologics where the 95% CI includes zero. The minimal importance difference for FEV1 is +/−150 mL [45, 46, 52, 54]. Benra, benralizumab; Dupi; dupilumab; Mepo, mepolizumab; Teze, tezepelumab.

For the subgroup of patients who had baseline eosinophils ≥ 300/μL, there were 5 H2H comparisons (Figure 9) [45, 46, 52, 54]. In one of the studies by Kim [54] there was a significantly greater improvement in FEV1 comparing Teze versus Benra, albeit with a wide 95% CI which excluded zero. One might predict Benra to exhibit greater efficacy than Teze for patients who have higher levels of eosinophils, but against that, airway smooth muscle has a high density of IL4/13 but not IL5 receptors [69].

Measuring effort‐independent low frequency respiratory impedance with oscillometry is more sensitive than FEV1 to detect changes in smaller airways < 2 mm in calibre from airway generations 8–23 [41]. In patients with a preserved FEV1 > 80% predicted, the presence of impaired R5–R20 predicts more frequent use of oral corticosteroid and salbutamol [70]. Moreover, abnormal values for R5–R20 and AX, but not FEV1, are associated with airway remodelling detected on high resolution CT scan [71].

**FIGURE 10:**
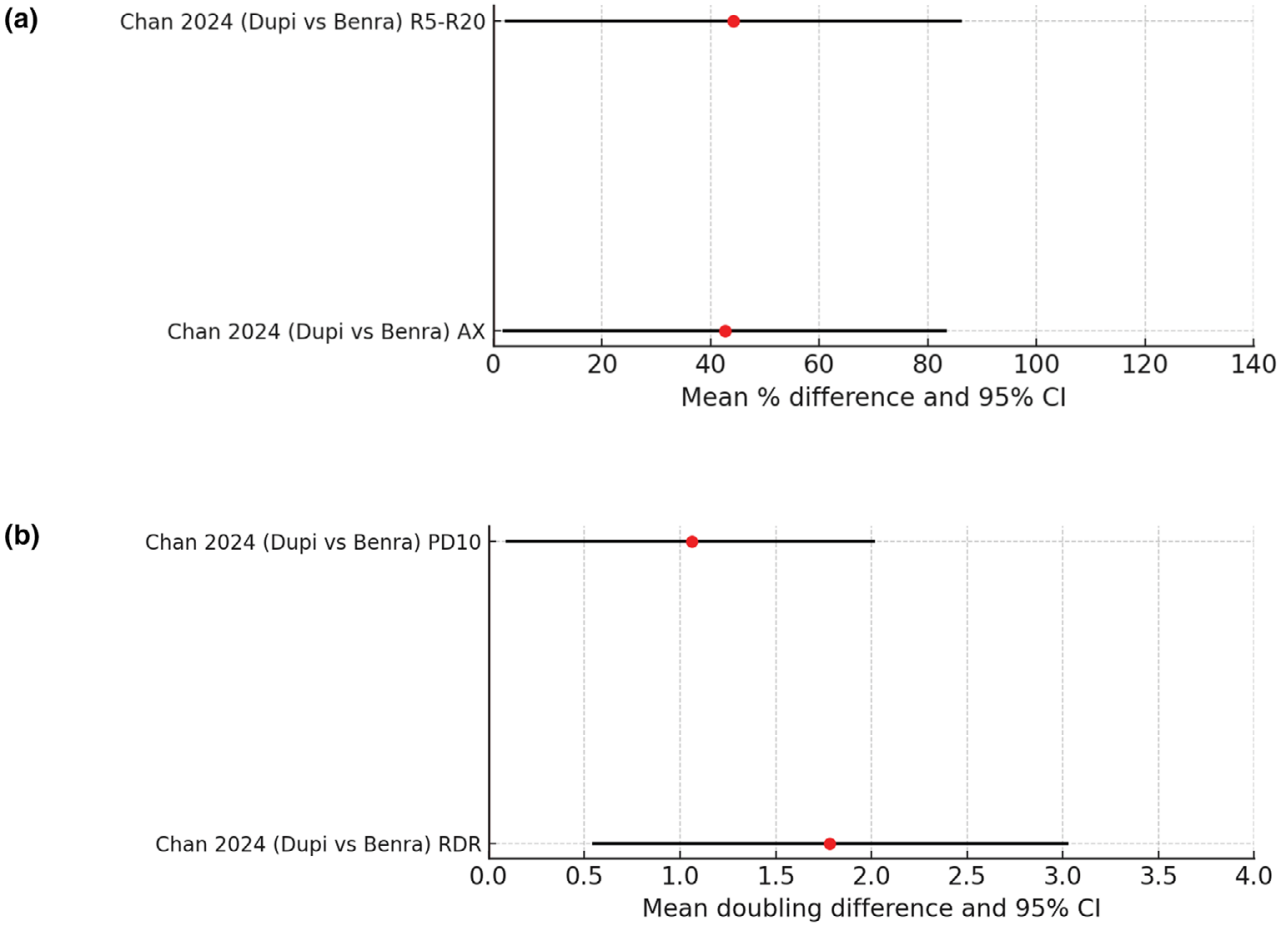
(a, b). Forest plot showing pairwise indirect comparisons of biologics in uncontrolled asthma for (a) improvements in AX and R5–R20 as mean % difference and 95% confidence intervals (CI) and (b) improvements in airway hyperresponsiveness as mannitol PD10 or RDR as mean doubling difference and 95% CI. For pairwise comparisons of drug A versus drug B, red circles denote a significant difference in favour of drug A where the 95% CI excludes zero [72, 73]. AX, Area under reactance curve; PD10, Challenge sensitivity as provocative dose of mannitol required to induce a 10% fall in FEV1; R5–R20, Resistance heterogeneity between 5 Hz and 20 Hz; RDR, Challenge reactivity as response dose ratio  for maximal % fall in FEV1 divided by the final cumulative mannitol dose.

A H2H case matched pairwise indirect comparison of Dupi versus Benra was analysed from oscillometry outcomes in two separate studies performed with a similar design from the same laboratory over 12 weeks in patients with T2 high severe asthma [72] (Figure 10a). Patients were selected on the basis of having oscillometry‐defined small airways dysfunction at baseline in terms of impaired peripheral lung resistance as R5–R20 ≥ 0.10 kPa/L/s and peripheral lung compliance as AX ≥ 1.0 kPa/L.

The two groups were well matched at baseline for mean R5–R20 with Dupi 0.22 kPa/L/s versus Benra 0.22 kPa/L/s and for mean AX with Dupi 4.62 kPa/L versus Benra 4.36 kPa/L, in turn indicating there was equivalent room for potential improvement with either drug. In response to treatment, the relative % improvements between Dupi versus Benra amounted to a difference of 44.2% (95% CI 2.1 to 86.3) for R5–20 and 42.6% (95% CI 1.7 to 83.5) for AX. While the 95% CI excluded zero, indicating a significant difference between drugs, the confidence intervals were wide. These apparent differences between drugs may be due to the opening up of the peripheral airways by the dissolution of mucus plugs as well as extensive expression of IL‐4/13 but not IL5 on smooth muscle in small airways [69, 74, 75]. In the study by Diver et al., no significant effects were observed on either R5–R20 or AX with tezepelumab compared to placebo, although patients were not selected a priori in regard to exhibiting oscillometry‐defined SAD.

## 9 Airway Hyperresponsiveness

Airway hyperresponsiveness (AHR) is a key tenet of persistent asthma and is clinically relevant in terms of representing the degree of bronchial twitchiness in response to exogenous stimuli [76]. Mannitol is an indirectly acting osmotic agent which induces AHR by lysing mucosal inflammatory cells such as eosinophils and mast cells [37, 77]. Two separate studies using the same design from the same laboratory were used in a case‐matched pairwise analysis to indirectly compare effects of Dupi and Benra for 12 weeks using mannitol AHR as the primary outcome to measure challenge sensitivity as the provocative dose to induce a 10% fall in FEV1 (PD10 as mg) or challenge reactivity as the response dose ratio which is the maximal % fall in FEV1 divided by the final cumulative mannitol dose (RDR as %/mg) [73] (Figure 10b).

For mannitol PD10, the Dupi and Benra patients were well matched with baseline geo mean PD10 values of 144 mg for Dupi versus 147 mg for Benra. The mean doubling difference in mannitol PD10 between Dupi and Benra amounted to 1.06 (95% CI 0.09 to 2.02) which was significantly different and also clinically relevant in terms of exceeding the MCID of +/−1.0 doubling difference [77] (Figure 10b).

Moreover, when defining patients who achieved remission of AHR after 12 weeks as a PD10 exceeding the maximal cumulative dose of 635 mg, there were 67% who met the criteria with Dupi versus 25% for Benra, which was also significantly different. For mannitol RDR, the mean doubling difference between Dupi and Benra was also significant at 1.78 (95% CI 0.54 to 3.03). Since Benra affords greater suppression of airway eosinophils than Dupi, this in turn suggests that the difference in AHR attenuation may be due in part to effects of Dupi on airway smooth muscle, where there is abundant expression of IL4/13 but not IL5, as demonstrated in ex vivo human small airways [69]. The greater effects of Dupi than Benra on AHR may be clinically relevant to patients who appreciate the feeling that their airways become less twitchy and consequently may not constrict in an unpredictable fashion in response to exogenous trigger factors. Although Teze has been found to also suppress mannitol AHR [18, 78] no formal indirect comparison against other biologics has been performed to date.

## 10 Conclusions

All biologics tend to be highly effective in patients with T2H uncontrolled severe asthma, and differences between them tend to be modest for key clinical efficacy outcome measures. In the present review, a synthesis of indirect comparative H2H studies between different biologics showed considerable heterogeneity for their effects on exacerbations, asthma control, quality of life and airflow obstruction. There was evidence from crude inspection of 95% CI for rate ratios that excluded unity to support greater reductions in overall AER with Dupi versus Mepo or Berna, and with Teze versus Berna, and in AER for Eos ≥ 300/μL with Teze, Dupi and Mepo versus Benra. There was also a greater reduction with Teze versus Dupi in AER for eosinophil < 150/μL. Overall, there were no differences between biologics for improvements in FEV1, but when categorised by eosinophils ≥ 300/μL, Teze was considered to be superior to Benra. Dupi was more effective than Berna for improving peripheral lung resistance and compliance, as well as for mannitol AHR. No differences were seen when comparing biologics for ACQ or AQLQ, where the effect sizes were small, along with widely overlapping 95% CI.

Prospective pragmatic RCT's are indicated to directly compare different biologics in uncontrolled type 2 high and low severe asthma patients. Such studies should be powered on the propensity of biologics to produce clinical remission. This, in turn, might well require collaboration between independent funding bodies and the pharmaceutical industry. A pooled reanalysis from data in existing phase 3 randomised controlled trials might usefully employ win ratios to indirectly compare biologics on a H2H basis looking at hierarchical composite end points, which have been employed in cardiovascular studies [79]. Prospective real‐life studies could also explore the possibility of different biologics producing complete clinical remission including outcomes such as inhaled corticosteroid sparing, abolition of AHR, small airways dysfunction using oscillometry, as well as attenuation of mucus plugs and remodelling as wall thickness on imaging.

Moving forward, bispecific biologics which block dual signalling pathways are in development, such as anti‐TSLP/anti‐IL13 nanobody (Sanofi, Lunsekimig) [63, 64], and it will therefore be important to know if such drugs confer any synergy of clinical response compared to mono‐specific blockers, in this case versus anti‐TSLP or anti‐IL13 agents.

## Author Contributions

All authors equally contributed to the manuscript with regard to the concept, writing and editing.

## Conflicts of Interest

Dr. Lipworth reports grants, personal fees (consulting, talks and advisory board), other support (attending meetings) from AstraZeneca; grants, personal fees (talks) and other support (attending meetings) from Sanofi and Regeneron, personal fees (talks and consulting) from Niox; grants, personal fees (consulting, talks, advisory board), other support (attending meetings) from Teva; personal fees (talks and consulting), grants and other support (attending meetings) from Chiesi; personal fees (consulting and talks) from Lupin, personal fees (consulting and talks) from Glenmark; personal fees (consulting) from Sandoz and Cipla; grants, personal fees (consulting, talks, advisory board), other support (attending meetings) from Boehringer Ingelheim; personal fees (consulting) from Bambusa and Upstream Bio), the son of BJL is presently an employee of AstraZeneca. Dr. Greig reports personal fees (talks) from AstraZeneca. Dr. Chan reports institutional grants from Chiesi and AstraZeneca; personal fees (talks and advisory boards) from AstraZeneca, Chiesi, Thorasys and Vitalograph; support for attending meetings from AstraZeneca, Chiesi, NIOX and Regeneron. Dr. Kuo reports personal fees from AstraZeneca, personal fees from Chiesi and non‐financial support from GSK outside the submitted work. Prof Jackson has shares as part of a managed portfolio in Verona Pharma, Canvatech PLC, Haleon PLC, GSK, Smith and Nephew, AstraZeneca, Hikma Pharmaceuticals, has a son currently working for AstraZeneca and is in receipt of an institutional grant from Chiesi.

## Data Availability

The data that support the findings of this study are available on request from the corresponding author. The data are not publicly available due to privacy or ethical restrictions.
